# Current Analytical Strategies in Studying Chromatin-Associated-Proteome (Chromatome)

**DOI:** 10.3390/molecules26216694

**Published:** 2021-11-05

**Authors:** Niamat Khan, Sidra Shahid, Abdul R. Asif

**Affiliations:** 1 Institute for Clinical Chemistry/UMG-Laboratories, University Medical Centre, Robert-Koch-Str. 40, 37075 Goettingen, Germany; niamat.khattak@gmail.com (N.K.); shahidsidra@yahoo.com (S.S.); 2 Department of Biotechnology & Genetic Engineering, Kohat University of Science and Technology, Kohat 26000, Khyber Pakhtunkhwa, Pakistan

**Keywords:** chromatin, euchromatin, heterochromatin, interphase, centromere, telomere, chromatin immunoprecipitation (ChIP), PICh, proteome, iTRAQ, QTIP

## Abstract

Chromatin is a dynamic structure comprising of DNA and proteins. Its unique nature not only help to pack the DNA tightly within the cell but also is pivotal in regulating gene expression DNA replication. Furthermore it also protects the DNA from being damaged. Various proteins are involved in making a specific complex within a chromatin and the knowledge about these interacting partners is helpful to enhance our understanding about the pathophysiology of various chromatin associated diseases. Moreover, it could also help us to identify new drug targets and design more effective remedies. Due to the existence of chromatin in different forms under various physiological conditions it is hard to develop a single strategy to study chromatin associated proteins under all conditions. In our current review, we tried to provide an overview and comparative analysis of the strategies currently adopted to capture the DNA bounded protein complexes and their mass spectrometric identification and quantification. Precise information about the protein partners and their function in the DNA-protein complexes is crucial to design new and more effective therapeutic molecules against chromatin associated diseases.

## 1. Introduction

Chromatin is a unique structure made up of proteins and nucleic acid which helps to tightly pack the nucleic acid within the eukaryotic nucleus. A number of cellular process, such as DNA packaging, transcriptional regulation, and DNA repair during cell divisions, are regulated by the chromatin. It exists in two predominant forms named as heterochromatin (condensed) and euchromatin (extended), respectively, by which it regulates the access of nucleic acid to various regulatory proteins and, thus, its cellular functions. Chromatin-associated proteins play a crucial role in accomplishing all these cellular activities [1]. However, to study these proteins is not trivial due to their low abundance in isolated chromatin complexes [2]. In addition, isolating chromatin complex itself is a laborious, time consuming, and expensive multistep process which is also prone to yield inconsistent results. Furthermore, these protocols are mostly developed for the chromatin-based study of higher eukaryotic cells and are not suitable for eukaryotic cells such as yeast [3]. 

Therefore, unbiased quantitative and qualitative proteomics strategies are indispensable to develop the structural and functional understanding to decode the underlying ‘Chromatome’ associated cellular activities [4]. However, to develop a single stand out strategy to address these questions is hard to develop mainly due to the existence of chromatin complex in different forms under various physiological and cellular condition. Here, we discussed various analytical strategies and highlight their importance in unraveling the information regarding chromatin associated proteins for better understanding of their role in various cellular functions. 

## 2. Isolation of Chromatome

**Figure 1:**
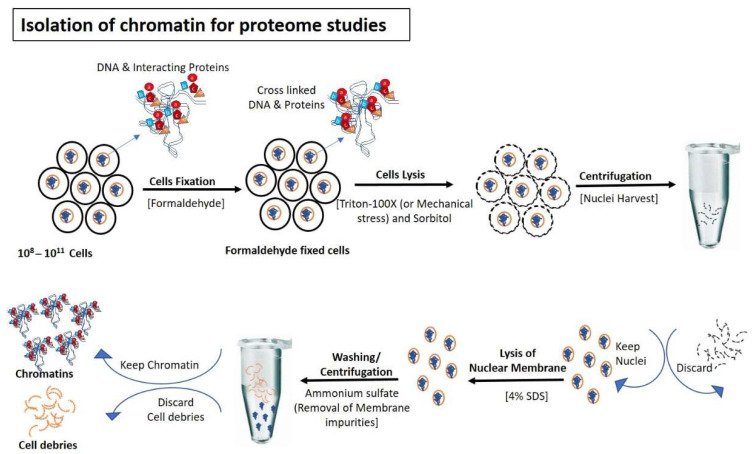
Isolation of chromatin for proteome studies: Cells are fixed normally with formaldehyde to avoid the detachment of DNA interacting proteins during the isolation steps of chromatin. After fixation, cells are lysed (using Triton-100×/mechanical stress) to rupture outermost boundary (Cell wall/plasma membrane) that nuclei remain intake to prevent contamination of cytoplasmic proteins. After centrifugation and removal of cytoplasmic contents, nuclear membranes are lysed with SDS and formaldehyde-mediated fixed chromatin are released in the buffer for further processing.

**Figure 2:**
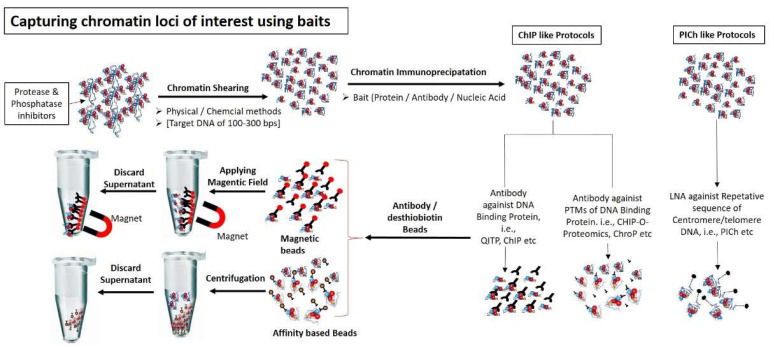
Capturing chromatin loci of interest using different baits: Chromatin are sheared into small fragments (100–300 bps) using either physical methods (i.e., sonication) or chemical method (i.e., Mnase enzyme). Chromatin specific fragments are captured using either protein baits (i.e., antibodies against DNA interacting proteins or against PTMs of interacting proteins) or nucleic acid probe (i.e., LNA, having specific sequence against DNA molecule present in chromatin fragments). Chromatin-bait complexes are precipitated using antibody binding beads (e.g., magnetic or streptavidin beads). Chromatin-antibody-protein beads complexes are washed and separated from non-specific unbound chromatin using magnetic field or appropriate column.

**Figure 3:**
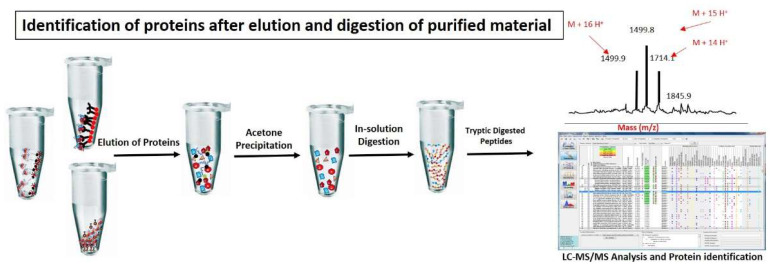
Identification of proteins after elution and digestion of purified material: Chromatin proteins are normally eluted from chromatin complexes using glycine or 3× Laemeli method. Eluted proteins are further purified by acetone precipitation method or SDS-page to remove excessive salts and trypsin digested. Tryptic digested peptides are further processed for identification and quantification of proteins through MS/MS analysis.

To isolate pure and high quality chromatin is a fundamental requirement to study chromatin associated proteins. However, to achieve this goal is a challenging task requiring high skills. A number of methods and kits to isolate chromatome are already available in the market and to discuss them in detail is beyond the scope of this review rather our focus is on analytical techniques used to analyze the chromatin associated proteins by mass spectrometry. However, to assist the reader, we provided a comparative list of important protocols for good quality chromatin isolation for mass spectrometry analysis in Table 1 and graphically illustrated in the Figure 1, Figure 2 and Figure 3.

## 3. Analytical Approaches in Chromatin-Associated Proteome

Chromatin structural organization various dramatically during different cellular states, such as “chromatin network”, i.e., a scattered and disorganized network in the interphase of resting cells to that of a highly condensed and well-organized thread-like structure known as “metaphase chromosomes” in dividing cells [13]. Similarly, chromosomes also have well distinct and diverse regions, such as the telomere, centromere, euchromatin (less condensed region and transcriptionally active), and heterochromatin that has more condensed and transcriptionally inactive or silent regions. Therefore, it is difficult to use a single strategy to study proteomes associated with different regions of chromatin and/or chromosomes. Proteomic scale study of chromatin demands a quick, simple and executable protocol that can enrich unbiasedly whole chromatin along with transiently bound factors [4]. A number of proteomic approaches and strategies have been developed or modified to investigate the functions of chromatin-associated proteins of a specific subset of chromatin. These are either locus-specific (i.e., PICh suitable for repetitive loci in the genome) or protein specific affinity (i.e., ChIP and their modified forms, based on antibody/affinity-tag-mediated precipitation of chromatin regions) [4]. Table 1 enlist the different proteomic approaches to study chromatin-associated proteins and to highlights the strategy gaps in the chromatin-associated proteomic approaches. Numerous tools have been used to analyze the chromatin proteomes of specific regions and/or conditions. These regions are retrieved by using a protein tag or specific antibodies against specific histone PTMs or DNA binding protein to immunoprecipitate chromatin fragments. In addition, specific complementary DNA sequences can be used as a bait to capture DNA-proteins complexes.

### 3.1. Chromatin Enrichment for Proteomics (ChEP)

Chromatin enrichment for proteomics (ChEP), is a simple biochemical method used to study the chromatin proteomics during interphase by enriching interphase chromatin in all its complexity. In contrast to exiting chromatin procedure, which focus on specific chromatin loci, ChEP enables us to analyze global chromatin composition along with its changes in response to various drug treatment or physiological conditions. 

ChEP has been successfully applied to present the first comprehensive inventory of human interphase chromatin [1]. Approximately 7635 human proteins have been identified that have a chromatin-based function. ChEP successfully precipitated around 3500 proteins [4]. In addition, ChEP is used to identify chicken chromatin proteins associated with cyclin-dependent kinase (Cdk) [4]. Apart from interphase, ChEP can also be used to purify mitotic chromosomes [4]. A number of factors can dwindle applications of ChEP in chromatin-associated proteome, such as inadequate crosslinking by formaldehyde (FH) and inefficient separation of chromatin-associated proteins from non-chromatin associated proteins.

#### 3.1.1. iTRAQ-Based Quantitative Interphase Associated Proteome

**Figure 4:**
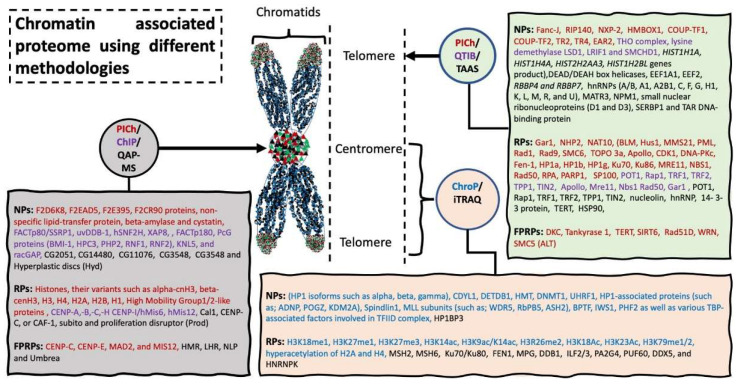
Chromatin associated proteome using different methodologies. Chromatin associated strategies are represented in circles, with arrow indicating the specific target location used for harvesting the sample material. Rectangle shaped represents novel proteins (NPs) and reported proteins (RPs) and protein previously reported but fail to be identified using these methodologies (FPRPs). Methodologies and their respective identified proteomics candidates are listed in same color. Abbreviation: Proteomics of isolated chromatin segments (PICh), tandem affinity associated strategy (TAAS), quantitative affinity purification-mass spectrometry (QAP-MS), chromatin immunoprecipitation (ChIP) (please see extended list in Abbreviations).

Isobaric tags for relative and absolute quantitation (iTRAQ) is an isobaric in vitro chemical labelling procedure to study quantitative proteomics by tandem MS [14,15]. A total of 681 proteins were quantified [5] and the study identified a number of factors that work in different complexes to maintain genomic integrity, e.g., increased MSH2 and MSH6 level was observed in heterochromatin of G1/S phase cells [5]. In addition, this study showed association of many transcription factors, such as ILF2/3, PA2G4, PUF60, DDX5, and HNRNPK, as well as transcriptional machinery proteins (POLR2A and POLR2B) with euchromatin [5]. Collectively, this iTRAQ based profiling study could identify 681 chromatin-associated proteins from different phases of the cell cycle, involved in genomic DNA, packaging, repair, and replication of genomic DNA and transcriptional regulation of many genes. Figure 4 lists the proteins identified using different strategies.

#### 3.1.2. CUT&RUN and greenCUT&RUN Methods

A recently developed “CUT&RUN” in situ method (cleavage under targets and release using nuclease) is used to conduct genome-wide profiling of transcription regulators, histone protein modifications, and chromatin-associated proteins [16,17]. CUT&RUN uses unfixed cells and antibody against MNase which is fused with protein A or G. This method does not use formaldehyde crosslinking and experience low background. It requires low cell numbers and very few read numbers (approximately 10%) due to a better signal-to-noise ratio, as compared to the ChIPseq method [17,18]. However, this method, like ChIPseq, still has drawbacks such as antibody specificity issues and PTMs of the epitope region which reduces antibody binding [19]. Another “greenCUT&RUN” method is used for genome-wide profiling of transcription regulators [19]. The method involves the use of GFP tagging of protein of interest followed by MNase fused GFP-nanobody for purification and mass spectrometric analysis to study genome-wide binding profiling [19]. This method eliminates the dependency on antibodies, uses GFP expressing cell lines, which is less time consuming, exhibits high resolution and reproducibility [19]. NFYA, FOS, and TBP transcription factors have been evaluated by this method, using known consensus motifs peaks for accuracy. NFYA interaction with SP1 and binding to DNA, FOS interaction with ATF/JUN family members, and TBP binding to TATA-box have been verified using this method [19].

### 3.2. Euchromatin and Promoter Associated Proteomic-ChIP-o-Proteomics

Epigenetic changes (such as DNA methylation, histones PTMs, and non-coding RNAs) play a crucial role to affect the regulation of gene expression [20]. Genes regulation are strongly associated with histones PTMs [21]. Number of studies reported that in eukaryotic cells, regulatory and coding regions of gene are strongly enriched with specific histone modification markers, such as active promoter region is decorated with H3K4me3, enhancer region with H3K27ac, and coding region with H3K79me2 and H3K36me3. In contrast, the promoter region of inactivated genes recruit H3K27me3 marker [6]. There are a number of other histones PTMs that are specific to the chromatin region during specific cellular activity. Immunoprecipitation using antibodies against these histones PTMs to purify the complexes has proved to be an effective tool to study the euchromatin associated proteome in specific state of the cell [20,22,23,24].

Promoter, the immediate upstream region (200–500 bps) of a gene, plays an important role in the regulation of gene expression [25]. Presence of the H3K4me3 histone marker allows trithorax group proteins to bind to promoter sequence and initiate transcription. The polycomb repressive complex 2 (PRC2) methylates H3K27 (H3K27me3) and inhibits the transcription [26,27]. These and several other histone marks (for details see review [28]) can be used to precipitate either DNA fragments of promoter regions and/or associated proteins to identify and/or confirm unknown and known factors in the promoter region of gene. Taking advantage of these histone markers, Khan and colleagues used ChIP-o-Proteomics approach to investigate promoter-associated proteins partners of the tight junction genes under the influence of immunosuppressive drugs (e.g., Mycophenolic acid, MPA) [29]). Study identified 333 proteins associated with active chromatin marker (H3K4me3), as well as 306 proteins associated with repressive chromatin marker (H3K27me3) of promoter regions. Quantification analysis of MS data revealed altered expression of 45 proteins precipitated with H3K4me3 antibody and seven proteins precipitated with H3K27me3 antibody [29,30]. Figure 4 presents a short overview of the chromatin associated proteins identified using different analytical approaches. The study shows the strength of the ChIP-o-Proteomics technique to investigate molecular mechanisms behind diseases and possible therapeutic agents to cure the disease. 

### 3.3. Heterochromatin Associated Proteomics

Heterochromatins are densely packed and silent regions of chromatin, which are further subdivided into two categories namely, constitutive heterochromatin and facultative heterochromatin. Genomic regions of constitutive heterochromatin remain the same, i.e., pericentromeric and telomeric, in every cell type of an organism and typically lack genes. Constitutive heterochromatin is structurally static and made of tandem repetitive DNA sequences known as satellites of various sizes starting from 5 base pair (bp) up to few hundred bps [31,32,33]. Epigenetic analysis identified a number of histone markers found in the heterochromatin region, such as H3K9me3, is described as a constitutive heterochromatin marker, and H3K27me3 is commonly found in facultative heterochromatin region [33]. A number of studies showed that these known markers and/or repetitive sequences could be used as baits to precipitate heterochromatin and to study associated proteins and/or DNA.

#### 3.3.1. Chromatin Protein (ChroP)

The ChroP method is designed to immunoprecipitate chromatin, similarly to previously established ChIP methodology, using constitutive histone mark (H3K9me3) and active transcription mark (H3K4me3) as baits, combined with quantitative proteomics using SILAC labelling to identify and quantify known and novel histone associated partners. ChroP effectively precipitates native chromatin using N-ChroP and formaldehyde treated chromatin using X-ChroP strategies. Native chromatin is sheared using micrococcal nuclease (MNase) having a DNA fragment size equal to one nucleosome while crosslinked chromatin via sonication having approximately DNA fragment size equal to two to three nucleosomes [14]. N-ChroP is suitable to study histone PTMs and X-ChroP to DNA-interacting and associated partners [34]. In parallel, precipitation of novel proteins, such as heterochromatin protein 1 (HP1 isoforms such as alpha, beta, gamma), CDYL1, DETDB1, HMT, DNMT1, UHRF1, HP1-associated proteins (such as ADNP, POGZ, KDM2A) at H3K9me3 advocate efficiency of ChroP methodology.

#### 3.3.2. “Middle Down”, “Top Down”, and Bottom-Up Approaches

Strategies to identify the PTMs of histones include “middle down”, “top down”, and bottom-up” approaches [35]. In the top down method, a complete panel of histone isoforms is detected by separating intact histones through chromatography and direct MS/MS-analysis [35]. Middle down approach uses residues of low frequency occurrence, such as Asp-N and Glu-C, cleaved by endoproteases, to obtain histone peptides of more than 5 kDa [35]. The top down and middle down approaches are also used in combination to study histone H3 and H2B [36]. Both these approaches detect long range PTM, however, these methods have low sensitivity and cannot be used to distinguish between peptides having same PTM on different positions [35]. Due to their requirement of high amount of starting material, they have limited use for the patient-derived samples. To overcome these shortcomings, bottom-up approach is used, which uses short peptides of 5–20 amino acid long. An Arg-C or Arg-C-like digestion method is used instead of trypsin which has low efficiency on core histones with high number of basic amino acids and therefore generate very short chains. Bottom-up approach has been successfully used on patient derived samples, however, it cannot provide sufficient information if more than four PTMs occur [35,36].

### 3.4. Telomere-Associated Proteomics

Quantitative telomeric chromatin isolation protocol (Q-TIP) allows comparison of telomeric-associated proteins of two cells having different telomeric states. QTIP is a combination of previously well-established analytical techniques such as SILAC labelling, ChIP and mass spectrometry. Antibody-mediated immunoprecipitation targets the telomeric proteins with high efficiency and specificity. QTIP mediated telomeric proteome of Hela cells has identified previously reported six abundant telomere-specific proteins, referred to as shelterin subunits [37,38], including POT1, Rap1, TRF1, TRF2, TPP1, TIN2, as well as other telomere-associated factors, such as Apollo, Mre11, Nbs1, Rad50, and Gar1. In addition, using QTIP new factors, such as THO complex, lysine demethylase LSD1, LRIF1, and SMCHD1 at telomeric region has been identified, which show proficiency of QTIP as analytical tool [7]. QTIP approach can be useful to investigate the disease models, such as cancer and could help to design targeted therapy to treat telomere associated fatal diseases.

Another approach is “Proteomics of isolated chromatin segments (PICh)” which is used to identify proteins associated with specific genomic loci of repetitive chromatin, such as telomeres. In PICh, inversely to ChIP, nucleic acid probe is used instead of immunoprecipitation. Nucleic acid probes consist of three components, such as locked nucleic acids (LNA), long spacer, and desthiobiotin. LNA probe, due to higher melting temperature as compared to the same DNA probe, facilitates strong interaction with DNA sequence (telomeric repetitive sequences), as well as stabilizes probe invasion. Long spacer, present between LNA and the immobilization tag, is used to reduce steric hindrance effects. Desthiobiotin works as an immobilization tag. Nucleic acid probe is hybridized to the target DNA moieties (such as; human telomeric repeats; (TTAGGG)n) that are present in formaldehyde crosslinked chromatin, followed by magnetic elution and identification of eluted proteins using MS. PICh efficacy was evaluated using three human cell lines (two telomerase positive Hela clones with different telomere length, and W138-VA13 ALT) [8]. PICh captured 210 proteins associated with Hela cell line telomeres and 190 proteins associated with ALT telomeres of W138-VA13 cells [8]. However, despite specific nucleotide probe complementary to specific sequence of telomeric region, PICh failed to precipitate and identify some already identified proteins, such as the Tankyrase 1 poly-ADP ribose polymerase, the Rad51D helicase, the Werner Syndrome helicase WRN, telomerase reverse transcriptase (TERT) and Dyskerin which indicates limitation of PICh in studying repetitive locus [8]. Therefore, further studies are needed to design efficient protocols suitable for the investigating the single locus proteome. 

### 3.5. Centromere-Associated Proteomic Strategies

The centromere, specialized chromosomal region, plays a crucial role in the accurate segregation of chromosomes during cell division (i.e., mitosis and meiosis) [39]. So far, 16 proteins have been identified in the CCAN further divided into five subcomplexes: the CENP-H/I/K/M, the CENP-L/N, CENP-O/P/Q/U/R, CENP-T/W/S/X complexes, and CENP-C [40,41]. Defects in centromeric-mediated regulatory pathways can alter segregation of chromosomes [42] that lead to a number of numerical chromosomal abnormalities, collectively known as aneuploidy. Aneuploidy is correlated with cancer and many genetic disorders [33,43,44]. Instead of resting cells, dividing cells provide an opportunity to capture and investigate centromeric-associated protein complexes, such as the kinetochore complex, that only bind when cells initiate the division process.

Unlike human, centromere of Drosophila melanogaster (D. mel) consists of only one additional constitutive centromeric protein, the conserved CCAN member; CENP-C. To analyze and identify the interacting partners of centromere, affinity-purified chromatin strategy was applied to D. mel cell line (Schneider S2 cell) that stably expressing either CID-GFP-tagged H3 variants or H3-variant-GFP, by MS. In total, 86 proteins that were specifically enriched in CENP-ACID chromatin were identified [11] that include known binding proteins such as Cal1, CENP-C, or CAF-1, subito and proliferation disruptor have previously reported to colocalize with centromere region [11,45,46,47]. Furthermore, loss of functions analysis showed that CG2051, CG14480, CAF-1 and hyperplastic discs are required for mitotic cell division [11]. However, on the other side, previously reported centromeric proteins, such as HMR, LHR, NLP, and Umbrea, were not detected by this strategy, indicating that further optimization is required to conduct comprehensive interactome of centromere.

#### 3.5.1. Centromeric Chromatin Associated Proteome Using PICh

Application of PICh technique identified a total of 94 proteins using Cen-probe, as well as 73 proteins by using Ser-probe. Among these proteins, 22 proteins were found common in both probes. Cen-probe mediated identified proteins are Histones, their variants, such as alpha-cnH3, beta-cenH3, H3, H4, H2A, H2B, H1, and high mobility group 1/2-like proteins [9,48]. Additionally, four proteins, with either predicted or unknown function, three proteins, non-specific lipid-transfer protein, beta-amylase and cystatin, were identified as a novel centromere partner. Nevertheless, PICh, like telomere locus, also failed to detect some previously characterized centromere partner possibly due to the fact that PICh requires large sample size. Application of MNase digestion for fragmentation of crosslinked chromatin could result in depletion of some proteins (e.g., CENP-A/B/C). Low efficiency of PICh hybridization, inefficient crosslinking could be the possible reasons of the partially failure of PICh mediated identification of centromere partners [9]. 

#### 3.5.2. ChIP Mediated Centromeric Proteome

Centromere-associated proteome was investigated using the ChIP strategy in the Hela cell line. Apart from known centromere proteins (CENP-H, CENP-I/hMis6, hMis12), 36 proteins were identified by proteomic analysis (e.g., FACTp80/SSRP1, uvDDB-1, hSNF2H, XAP8, FACTp180, PcG proteins) many of them with previously unknown functions. Furthermore, cytological localization analysis reveals presence of uvDDB-1 in centromere throughout the cell cycle, while BMI-1 remains associated with centromere only during interphase. Functional analysis of the identified partners of centromere with unknown function will help to understand the molecular architecture of centromere [12] and molecular mechanism behind centromere-associated diseases.

### 3.6. Non-Replicative Chromatin-Associated Proteome, aniFOUND Approach

Accelerated native isolation of factors on unscheduled nascent DNA (aniFOUND) is a antibody-free method that can be applied to capture nascent chromatin fragments synthesizing (outside S-phase of cell cycle) due to specific cues, such as UV irradiation. This unscheduled DNA synthesis (UDS) often occur as a repairing mechanism of UV-induced DNA lesions. aniFOUND was developed based on nucleotide excision repair (NER) mechanisms and click chemistry-based protocols [49]. aniFOUND has been successfully tested in immortalized human skin fibroblast cell lines (1BR.3, VH10 normal, and NER-deficient XPA hTert) exposed to UVC irradiation. MS analysis reveals identification of 323 proteins and their association with UVC-mediated UDS, termed as UVC-UDS’ome. The antiFOUND strategy can be used to investigate the pathomechanism of NER linked cancers and rare human disorders, such as Cockayne syndrome, trichothiodystrophy, and xeroderma pigmentosum [50,51,52].

### 3.7. G4-Quadruplex Associated Proteomic Approach Using CMPP Strategy

The human genome consists of more than 700,000 sites, consisting of non-canonical, four-stranded nucleic acid structures that are enriched in G sequences, known as G-quadruplexes (G4s) [53,54]. These are dynamic structures present in active promoters of certain genes (such as cancer genes) that are highly expressed [55]. Many proteins (i.e., enzymes, transcription factors) have been identified using synthetic G4 oligonucleotide baits, to interact with G4s sites in vitro studies (detail in [56]). Recently, co-binding-mediated protein profiling (CMPP) strategy was introduced to investigate G4s interacting proteins in native chromatin [56]. Label-free quantitative LC MS/MS proteomics approach revealed that both probes captured significant number of proteins bind to G4 site (probe I capture 248 proteins and probe II capture 209). Remarkably, 201 out of 209 (approximately 96%) probe II captured proteins were also found in probe I enlisted proteins. Among these identified proteins, 24% proteins captured by probe I and 14% protein captured by probe II matched with previously identified proteins. These findings confirm the accuracy of CMPP mediated target proteins identification, associated with G4 sites. [56]. Further CMPP mediated studies of highly expressed gene promoter will help us to identify cancer associated novel factors.

## 4. Conclusions

Unlike genome, proteome is a diverse and dynamic subject to the cellular activities as well as the environment. Although, traditionally available methods can functionally characterize individual locus specific proteins but these methods are very labor intensive, expenses, and show poor yield. Therefore, high-throughput proteomic techniques are required for the large scale profiling of locus specific proteome. In this review article, we focus on strategies that are currently applied to investigate chromatin proteome in resting and dividing cells. Due to the dynamic and well-organized structure of chromatin, contamination free specific chromatin fragments isolation is only possible using specialized protocols and training. The authors anticipate an increase interest in this area in the coming days due to its importance to finding novel disease markers and drug targets. Strategies such as QAP-MS, ChIP, PICh (reviewed in the article) have precipitated in identification of approximately 2100 centromere associated proteins in different organisms. ChIP-O-Proteomics have identified more than 300 proteins associated with histones specific PTMs markers in active and or repressive promoter region. Functional analysis of the identified proteins and identification of new proteins associated with chromatome will help to understand the mechanism behind the proliferative diseases and identify new therapeutic targets. 

## Figures and Tables

**Table 1:** Approaches to identify specific chromatin locus associated proteome.

Technique	Material			Chromatin Shearing/Isolation			Identified Proteins	Ref.
	Cells	Fixation	Lysis Buffer	Chromosomes (Region)	Shearing Approach	Bait		
iTRAQ-ChEP	293F Cells	NA/Nonidet P-40	10 mM KCl, 10 mM HEPES pH 7.5, 1 mM DTT, 1 mM MgCl2, 0.34 M sucrose, 0.1% triton X-100, and protease inhibitors	Chromatin	MNase, 5–10 U, 37 °C, 10 min		481	[5]
ChroP	HeLa S3 Cells	0.75% FH	60 mM KCl, 15 mM NaCl, 15 mM HEPES, 10% sucrose, 1 mM DTT, 0.5 mM EGTA pH 8.0, 0.5 mM PMSF, 0.5% Triton, 5 μg/mL Aprotinin, 5 μg/mL Leupeptin.5 mM NAF, 5 mM Na3VO4, 5 mM NaButyrate, 5 μg/mL Pepstatin A.	(Hetero and Euo) Chromatin	MNase: 0.005 U/L, 37 °C, 60 min Sonication: Power 200 W, 15 min (cycles of 30 s on/1 min off)	Anti- H3K9me3	837	[6]
						Anti- H3K4me3	381	
QTIP	HeLa Cells	1% FH	50 mM Tris-HCl pH 8.0, 10 mM EDTA-NaOH pH 8.0, 1% SDS, EDTA-free protease inhibitors	Telomere	Sonication Bioruptor: 6 °C, (30–40 Cycles, 30 s on/off) Focused-Ultrasonicator (E220, Covaris, duty: 5.0, PIP: 140, cycles, 4 °C, 30 min	Anti-TRF1, and Anti-TRF2	324	[7]
PICh	HeLa S3 Cells	3% FH	LBJD: 10 mM, HEPES-NaOH, pH 7.9; 100 mM NaCl; 2 mM EDTA, pH 8; 1 mM EGTA, pH 8; 0.2% SDS; 0.1% Sarkosyl, protease inhibitors LBJDS: 0 mM, HEPES-NaOH, pH 7.9; 30 mM NaCl; 2 mM EDTA, pH 8; 1 mM EGTA, pH 8;0.2% SDS; 0.1% Sarkosyl, Protease inhibitors	Telomere	Sonication: Power setting 7 (36–45 Watts), 15 s pulse on, 45 s pause off, 7 min	nucleic acid probe	210 (Hela)190 (W138-VA13)	[8]
		3% FH	100 mM NaCl,10 mM Potassium Phosphate pH 7.0, 12% Hexylene glycol, 0.1% mercaptoethanol	Centromere	MNase: 0.15 U/1 ug Chromatin, 37 °C, 10 min Sonication: 10% power out, 8 cycles (15 s on, 45 s off)	Cen-probeor Ser-probe)	94 (Cen-probe) 73 (Ser-probe)	[9]
Tandem Affinity Associated Strategy (TAAS)	293T cells	1% FH	50 mM Tris-HCl, pH 8.0, 150 mM NaCl, 1 mM EDTA, pH 8.0, 1% Triton X-100, 0.1% SDS, and freshly added protease inhibitors: 1 μg/mL aprotinin, 1 μg/mL leupeptin, 1 μg/mL pepstatin, 1 mM phenylmethylsulfonyl fluoride	Telomere	Sonication: Six times, 30 s on, Misonix Sonicator 3000 (in between). 30-s rest	HA-FLAG-TIN2 Or HA-FLAG-GFP	92	[10]
Quantitative affinity purification-mass spectrometry (QAP-MS)	Drosophila Schneider S2 cell line		15 mM NaCl, 1.5 mM MgCl2, 1 mM DTT, 0.1 mM EDTA, 0.1% Triton X-100, 0 mM HEPES pH 7.6, Protease inhibitors	Centromere	MNase: 2000 U/10^9^ cells, 20 min, 26 °C	Anti-GFP Affinity	1871, 86 (CENP-A^CID^ chromatin)	[11]
ChIP	Hela S3 cell line (5 × 10^9^ cells)		20 mM Hepes, pH 8.0, 15% (*vol*/*vol*) glycerol, 03 mM PMSF, 1 mM NaHSO3, 0.5 mM EDTA, 0.5 mM DTT, 2μg/mL leupeptin and 0.5 μg/mL pepstatin.	Centromere	Mnase: 6 U/mL Time = 30 min. Temperature = 37 °C	anti-CENP-A	40	[12]

## Data Availability

Not applicable.

## Abbreviations

Abr.	Full Name	Abr.	Full Name
ADNP	Activity-dependent neuroprotective protein	MARs	Matrix attachment regions
ASH2	*Ash2* histone methyltransferase complex subunit *ASH2*	MCF-10A	*MCF 10A* cell line is a non-tumorigenic epithelial cell line
BPTF	bromodomain PHD finger transcription factor	MCF-7	Michigan Cancer Foundation-7
BT-474	breast tumor *cell line BT*-*474*	MDA-MB-453	*MDA*-*MB*-*453* is a human breast cancer cell line
BT-549	human breast cancer *cell line BT549*	MDA-MB-468	MD Anderson-Metastatic Breast-468
CCAN	constitute centromere-associated network	MLL	Mixed Lineage Leukemia (MLL) Protein
CDYL1	Chromodomain protein Y-like 1	MNase	Micrococcal nuclease
CENP-C, or CAF-1	Centromere Protein C, Chromatin assembly factor-1	MPG	human N-methylpurine–DNA glycosylase (MPG) gene
ChEP	Chromatin enrichment for proteomics	Mre11	Meioticrecombination 11
CUT&RUN	cleavage under targets and release using nuclease	mRNA	MessengerRNA
DDB1	Damaged DNA binding protein 1	MS	Mass spectrometry
DDX5	DEAD-Box Helicase 5	MSH2	MutS homolog 2
DNA	Deoxyribonucleic acid	MSH6	MutS homolog 6
DSBs	double strands breaks	Nbs1	Nijmegen breakage syndrome 1 (*Nbs1*)
FACTp80	Facilitates Chromatin Transcription 80 kD subunit	NHEJ	Non-homologous end joining
FEN1	Flap endonuclease 1	PA2G4	Proliferation-associated protein 2G4
G1, S, G2	Gap 1, Synthesis, Gap 2	PcG proteins	Poly comb group *proteins*
H1	Histone 1	PHF2	Plant homeodomain finger 2
H2A	Histone *H2A*	PHP2	Pseudohypoparathyroidism type 2
H3	Histone 3	PK	Proteinase k
HBL-100	*HBL*-*100* is an epithelial *cell line*	POGZ	Pogo Transposable Element Derived With ZNF Domain
HCC1954	*HCC1954* is positive for the epithelial *cell*	POT1	Protection of telomeres protein 1
HEK293	primary embryonic human kidney (HEK293),	PTMs	Post Translational Modifications
HeLa	human cells derived from cervical cancer	PUF60	Poly-binding-splicing factor PUF60
HepG2	human liver cancer cell line	QqTOF	quadrupole-quadrupole-time-of-flight (*QqTOF*)
HNRNPK	Heterogeneous nuclear ribonucleoprotein K	QTIP	Quantitative Telomeric Chromatin Isolation Protocol
HP1	Heterochromatin Protein 1	racGAP	Rac GTPase-activating protein 1
HPC3	Hereditary Prostate Cancer 3	Rap1	Ras-proximate-1
HR	homologous recombination	RFC4	Replication factor C subunit 4
Hs578T	breast cancer *cell line*. *HS578T*	rRNA	Ribosomal RNA
hSNF2H	Sucrose nonfermenting protein 2 homolog	SDS-PAGE	sodium dodecyl sulphate-polyacrylamide gel electrophoresis
Hsp 70	70 kilodalton heat shock proteins	SK-BR-3	SK-BR-3 is a human breast cancer cell line
ILF2	Interleukin Enhancer Binding Factor 2	SMCHD1	Structural Maintenance of Chromosomes flexible Hinge Domain Containing 1
ILF3	Interleukin enhancer-binding factor 3	SUM1315MO2	Cellosaurus *SUM1315MO2 cell line*
iMDK	midkine inhibitor	SUM159PT	Cellosaurus SUM159PT cell line
iTRAQ	Isobaric tags for relative and absolute quantitation	T-47D	*T*-*47D* is a human breast cancer *cell line,*
KDM2A	Lysine-specific demethylase 2A	TIN2	TERF1-interacting nuclear factor 2
Ku70/Ku80	Ku is a dimeric protein complex of DNA repair.	TPP1	Tripeptidyl Peptidase 1
LCRs	Locus control regions	TRF1/TRF2	Telomeric repeat-binding factor 1 and 2
LNA	Locked Nucleic Acids	tRNA	Transfer RNA
LRIF1	Ligand Dependent Nuclear Receptor Interacting Factor 1	U2OS	osteosarcoma *U2OS cell line*
LSD1	Lysinedemethylase 1	uvDDB-1	Ultraviolet DNA damage binding protein 1
LY2	*LY2* breast cancer cells	ZR-75-1 cell line	*ZR*-*75*-*1* is a human breast cancer *cell line*
